# On the pursuit of the true impact of our actions in science

**DOI:** 10.3389/frma.2026.1783120

**Published:** 2026-05-05

**Authors:** Massyel S. Martínez-Cortés, José L. Medina-Franco

**Affiliations:** DIFACQUIM Research Group, Department of Pharmacy, School of Chemistry, Universidad Nacional Autónoma de México, Mexico City, Mexico

**Keywords:** accomplishment, chemoinformatics, computer-aided drug design, impact factor, metric, science practitioners, time, wellbeing

## 1 Introduction

As practitioners in the chemical sciences, we frequently simplify the systems under study, for example, through data reductionism (Maggiora, 2011, 2022). This is because understanding complex systems, with all known and unknown variables, can become quite difficult and, more realistically, nearly impossible to achieve. For example, in basic research in computer-aided drug discovery or, as Dr. Gisbert Schneider calls it, computer-aided bioactive compound discovery, one isolates chemical compounds and their molecular targets as an extreme approach to design what we expect will eventually become a drug. In such a process of “rational drug design” (Saldivar-González et al., 2023), we assume an extremely large number of other variables and want to think that the compounds will become a drug and help to cure a disease. While this reductionist approach is convenient for tackling complex problems and advancing basic science, it is also coarse, and scientists are not always fully aware of the many assumptions involved, often losing track of relevant variables that are difficult to control.

With the rapid advances in data availability and artificial intelligence (AI) in science, including drug discovery (Jiménez-Luna et al., 2021; Jacobson, 2025; Zhang et al., 2025), a larger number of variables are being taken into account, and we have the sense that we are in better control of complicated systems. However, there is also considerable hype around the idea that AI may independently design drugs. In reality, many of these algorithms operate as “black boxes,” limiting our ability as human researchers to fully rationalize their outputs, despite ongoing efforts in explainable AI (Lavecchia, 2025).

Indeed, as students, postdocs, and research investigators—hereafter referred to as science practitioners (SPs)—we are inherently trained to simplify variables. In chemoinformatics, defined as the use of informatics to solve chemical problems (Gasteiger, 2016; Bajorath, 2024), these simplified variables, or “descriptors,” underpin computational models and define the so-called “chemical space” (Reymond, 2025), which can expand into multiple “chemical multiverse” depending on research goals (Medina-Franco et al., 2022).

In any case, SPs, from early stages of their development, seek to contribute meaningfully to their communities and surroundings. Throughout their academic and professional trajectories, they are evaluated through grades, metrics, and numerical data intended to quantify performance. Tensions arise when these metrics interfere with personal and professional goals as human beings. In this context, this manuscript aims to reflect on how SPs strive to generate real impact through scientific actions beyond such metrics. This reflection is guided by analogies between life and chemistry research, particularly within chemoinformatics, one of the main research areas of the authors.

## 2 Goals: the cornerstone of research projects

A cornerstone of any research project is the objective; it addresses a direct question: what do we want to accomplish? In any research proposal, thesis, report, scientific paper, or conference presentation, the SP must define a main goal and, frequently, specific goals, which are planned steps that help to reach the main goal. Clearly, identifying and writing down such goals is not trivial and may take considerable time. However, it is necessary because the main goal guides all research steps, including how time, energy, and economic resources are spent. It is not rare—indeed, it is fairly frequent—that goals, mainly specific ones, need to be adjusted as research progresses. New findings and unexpected positive or negative results shape the path toward the main objective.

## 3 Life goals

As human beings, SPs also need to define goals in their personal lives, both main and specific. Over time, such goals evolve and change as we grow and move on with our lives. As in research projects, goals are shaped by the positive and negative experiences we all face. In analogy with scientific practice, defining personal and professional goals is not trivial, mostly because, in the end, it is our own responsibility and we must live with the outcomes of our decisions.

### 3.1 Defining a life goal or life purpose: living in multiverses

As in science, defining individual goals as human beings is not straightforward; in fact, it is often more complex than defining research objectives. In this process, we also rely on a form of reductionism, as considering all variables in life simultaneously can be overwhelming. Life goals are dynamic and evolve over time alongside the variables that shape them, such as personal preferences, economic needs, education, job stability, and interpersonal relationships. These factors continuously change, giving rise to what can be described as a “life space,” which, much like chemical space in chemoinformatics, evolves over time. In this sense, individuals experience a kind of “life multiverse,” where the variables driving decisions and goals are constantly shifting.

### 3.2 Finding the sweet spot and consensus goals: science practitioners as humans

As discussed above, SPs, must define both personal and professional goals. However, academic practice is often strongly influenced by the pursuit of “impact,” which is typically quantified through metrics developed by the scientific community, each with advantages and disadvantages (University of Illinois Chicago, 2026a,b; Akhtar, 2024). All such metrics, reviewed in detail elsewhere, are imperfect and remain under constant discussion, refinement, and revision (Bornmann and Marx, 2016; Jemielniak, 2025). Metrics are necessary, as science is driven by quantification. As Dr. Jonathan Goodman once said in a class: “If you understand a (scientific) concept, you can quantify it.” Nevertheless, an overemphasis on quantification may lead SPs to conflate metrics, such as impact factors or citation counts, with the true purpose of research (Medina-Franco and López-López, 2022; Liu and He, 2023).

This issue becomes particularly evident when students and researchers are encouraged to “make an impact in science,” often interpreted as publishing in high-impact journals or accumulating citations. In such cases, scientific practice may shift toward the pursuit of metrics rather than meaningful contribution. Although grades and metrics are valuable, important, and often motivating, they should not become the primary objective of scientific activity (Meho, 2025).

In the context of drug discovery, the question of impact becomes even more relevant. Is the impact of a research project defined by the journal in which it is published, its visibility on social media, or the number of citations it accumulates? While these indicators can be useful, they should not define the value of a project, the effort invested, or the wellbeing of researchers. Ultimately, the focus of drug discovery is the human being, and most beneficiaries of scientific advances are neither aware of nor influenced by publication metrics, yet they benefit directly from scientific work (Ali and Djalilian, 2023).

## 4 When true progress is not the final outcome

In both life and science, success is often associated with visible and quantifiable outcomes. In everyday life, this may include material possessions or financial achievements, while in drug discovery, it may involve identifying candidate molecules, publishing papers, or completing clinical trials. However, this view overlooks a fundamental part of the process. At times, the actions carried out day by day—often imperceptible yet ultimately impactful—are not recognized by others (including evaluation systems) as successes.

The process of searching for a new drug resembles learning how to navigate more than reaching a destination directly. Numerous hypotheses are tested, many of which do not yield the expected results. Promising compounds are discarded, and models are continuously refined. Each step, even those that appear unsuccessful, contributes to a deeper understanding of the problem and helps delineate future directions. Similarly, in personal and professional life, decisions that do not immediately lead to success often provide valuable insights into preferences, limitations, and priorities.

What is often perceived as failure may, in fact, represent meaningful progress. In drug design, for example, a modification that reduces binding affinity can still provide critical information about molecular interactions, model limitations, or experimental constraints. Progress lies in the ability to extract knowledge from such outcomes and use it to refine future approaches. Life follows a similar pattern, where changes in direction and reevaluation of goals are not setbacks but essential components of growth.

Although only a small fraction of compounds ultimately become drugs, each research effort contributes to collective knowledge by improving predictive models and informing future strategies. In the same way, not all personal or professional efforts lead directly to desired outcomes, yet they contribute to clarity, judgment, and maturity. Recognizing that progress is not solely defined by outcomes allows for a broader and more meaningful understanding of impact, both in science and in life.

## 5 Summary conclusions

SPs are individuals whose scientific and personal goals evolve over time in response to diverse experiences. Throughout their training and careers, metrics are used to quantify performance and assess impact. While these metrics serve an important purpose, they can misguide scientific practice when they become the primary objective.

Our main conclusion is an invitation to readers—peers, students, and SPs—to reflect on questions such as: What goals have guided their decisions? What variables or factors have guided my past and present goals (i.e., what is my Life Space)? Which of these variables are fixed, and which are flexible?

Ultimately, we encourage SPs to identify the true impact of their work, recognizing that while metrics are important, they should not define scientific practice or personal fulfillment. Science is not only about outcomes but also about the process, the questions we ask, and the continuous pursuit of understanding, elements that are inherently difficult to quantify, yet central to meaningful progress.
